# Construction and implementation of wide range parameter switchable chaotic system

**DOI:** 10.1038/s41598-024-54458-2

**Published:** 2024-02-19

**Authors:** Minxiu Yan, Xindi Liu, Jingfeng Jie, Yue Hong

**Affiliations:** School of Information Engineering, Shenyang University of Chemical Technology, Shenyang, 110142 China

**Keywords:** Chaotic system, Switching method, Unified chaotic system, Multisim circuit simulation, Physical implementation, Mathematics and computing, Physics

## Abstract

Research on switchable chaotic systems with a large range of parameters is scarce. To explore the chaotic characteristics of such systems, this paper proposes new switchable methods by modifying the nonlinear term in the system, resulting in a chaotic system with different nonlinear terms. The unknown parameters in the nonlinear term exhibit different numerical relationships under various combined conditions, and some parameters may tend towards positive infinity. The chaos characteristics are verified by applying a specific switching method to the unified chaotic system. The pseudo-randomness of the random sequence generated by the dissipative system is verified using the NIST test. Finally, the circuit simulation of the system under various switching conditions is performed by selecting different circuit components and adjusting the resistance values.The switching chaotic system is implemented physically on FPGA and breadboard, and the effectiveness of the system is verified.

## Introduction

In recent years, chaotic systems with different order^1–4^ chaotic attractors and various switching chaotic systems^5–7^ have chaotic characteristics, which have attracted wide attention in various fields. Due to the uncertainty of the switched system, it usually shows the coexistence of multiple scrolls^8^ or multiple periodic attractors^9^ of the chaotic system under the same initial condition^10^. Therefore, switchable chaotic systems have more complex topologies, which makes chaotic systems of great value in security fields such as secure communication^11^.

Many scholars have achieved fruitful results in exploring chaotic systems, the application and synchronization of parameter switching systems and switching systems have also been developed^12,13^. Zhang et al.^14^ successfully extended the Hilnikov criterion to the switching system, and obtained the feasible method of the heteroclinic orbit according to the heteroclinic loop criterion.Nitish et al.^15^ designed a controller that could achieve synchronization in a variety of complex states, and realized the synchronization of multiple non-homogeneous systems. It could produce more complex and rich dynamic evolution than a single drive system. Due to the powerful computing power and high flexibility of FPGA, it is often used as a hardware implementation platform for chaotic systems. Dong et al.^16^ used sine function to construct a controllable multivortex conservative chaotic system, and implementing the system under FPGA^17^. In practical application, due to device error and other reasons, the simulation results cannot be guaranteed to be consistent with the actual experiment. Therefore, it is necessary to verify the physical circuit of the chaotic system. Gong et al.^18^ used breadboard physically implemented a four-dimensional chaotic system. The physical realization of complex and diverse chaotic systems and the verification of the feasibility and effectiveness of the systems are of great significance to the application of chaos in various fields^19^.

Inspired by the previous literature, this paper studies a new method to adjust the nonlinear term of the system. Different switching forms will lead to changes in the dynamic behavior of the system. It is verified that the nonlinear parameters of the system under the switching term are close to positive infinity. In order to verify the feasibility of the proposed switching chaotic system, the circuit simulation and physical implementation of the system are carried out in this paper. The research results of this paper provide ideas and methods for the application of switching chaotic systems, and have potential application value in the fields of information encryption^20,21^.

## Dynamics analysis of chaotic system

### System model and equilibrium poin

Based on the classical Lorenz system, the system model is obtained by modifying the nonlinear term and unknown parameters:

1 $$\begin{aligned} \left\{ \begin{array}{lll} \dot{x}=-ax+yz\\ \dot{y}=bx-xz\\ \dot{z}=-z+xy+c \end{array} \right. \end{aligned}$$

**Figure 1:**
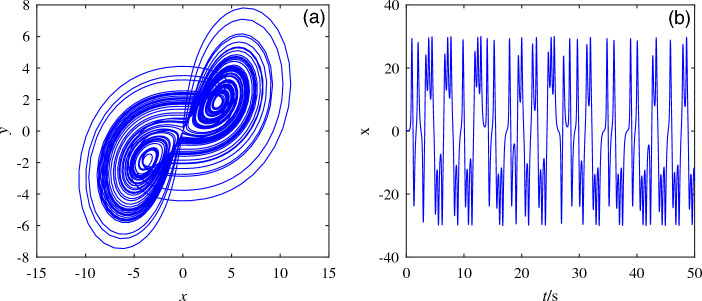
Attractor and timing diagram. (**a**) Attractors with unknown parameters $a = 5, b = 8, c = 1$. (**b**) Time series diagram.

Where system (1) contains seven terms and a constant term, *x*, *y*, *z* are the state variables of the system and *a*, *b*, *c* are the unknown parameters of the system. When the unknown parameters are $a = 5, b = 8, c = 1$ and the initial values are (0.1, 0.1, 0.1), the attractor diagram and the timing diagram are shown in Fig. 1.

System (1) remains unchanged under the coordinate transformation of $(x,y,z)\rightarrow (-x,-y,z)$, and the system is rotationally symmetric for *z* axis.

By calculating the dissipation degree of system (1), the dissipation degree $\nabla {V}$ can be obtained:

2 $$\begin{aligned} \nabla {V}=\frac{\partial {\dot{x}}}{\partial {x}}+\frac{\partial {\dot{y}}}{\partial {y}}+\frac{\partial {\dot{z}}}{\partial {z}}=-a-1 \end{aligned}$$

Equation (2) shows that $\nabla {V}<0$ and unknown parameter is $a>-1$, system (1) is a dissipative system. At this time, the system is a three-dimensional nonlinear dynamic system. The trajectory of the system will shrink and fold according to the negative exponential rate of $V(0)e^{-\delta {t}}=V(0)e^{-6t}$, and finally will be in an invariant attractor set.

Analyze the equilibrium point of system (1), set the left side of the equation equal to zero, and solve the equilibrium point of system $E_1$, $E_2$ and $E_3$. The stability of the equilibrium point was analyzed according to the Routh-Hurwitz stability criterion, and the eigenroots corresponding to the equilibrium point of the system were obtained, as shown in Table 1.

**Table 1:** System equilibrium stability.

Equilibrium points	Characteristic roots	Equilibrium type
$E_1=(0,0,1)$	$\lambda _{11}=1.14$, $\lambda _{12}=-1$, $\lambda _{13}=-6.14$;	The saddle-focus equilibrium point with index 1
$E_2=(\frac{\sqrt{a(b-c)}}{a},\frac{\sqrt{a(b-c)}}{b},b)$	$\lambda _{21}=-6.72$, $\lambda _{22}=0.36-4.07i$, $\lambda _{23}=0.36+4.07i$;	The saddle-focus equilibrium point with index 2
$E_3=(-\frac{\sqrt{a(b-c)}}{a},$ $-\frac{\sqrt{a(b-c)}}{b},b)$	$\lambda _{31}=-6.72$, $\lambda _{32}=0.36-4.07i$, $\lambda _{33}=0.36+4.07i$;	The saddle-focus equilibrium point with index 2

### The Lyapunov exponent, dimension and power spectrum

**Figure 2:**
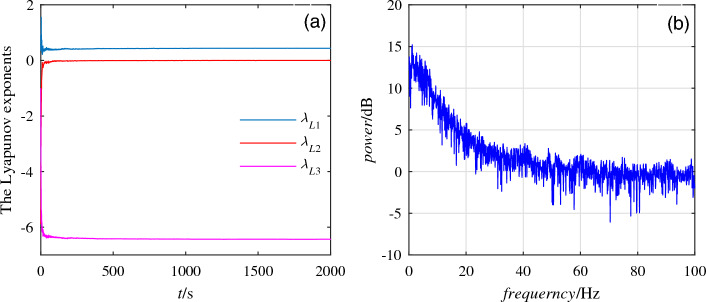
The Lyapunov exponent diagram and power spectrum diagram. (**a**) The Lyapunov exponent diagram for unknown parameters $a = 5, b = 8, c = 1$. (**b**) Power spectrum.

When the unknown parameters are selected as $a = 5, b = 8, c = 1$ and the initial values are (0.1, 0.1, 0.1), the Lyapunov exponent of system (1) are $\lambda _{L1}=0.4306,\lambda _{L2}=0,\lambda _{L3}=-6.4349$ as shown in Fig. 2a. Fig. 2b shows the power spectrum of the system is continuous and there is no obvious peak.

Under the premise of selecting the initial conditions, the Lyapunov exponent of system (1) in Fig. 2a contains positive real numbers and the Lyapunov dimension satisfies the condition of fractional dimension, indicating that system (1) exhibits chaotic properties. In Fig. 2b, the power spectrum image of system (1) is continuous and there are no obvious spikes, indicating that system (1) contains chaotic attractors, further proving the chaotic nature of system (1).

### System dynamics analysis under variable parameters

**Figure 3:**
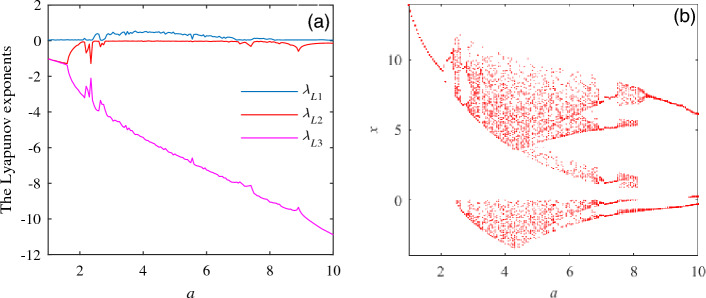
The Lyapunov exponent diagram and bifurcation diagram. (**a**) The Lyapunov exponent diagram for *b* = 8, *c* = 1. (**b**) Bifurcation diagram with $b = 8, c = 1$.

To further explore the chaotic characteristics of the system, unknown parameters are selected $a\in {[1,10]}, b = 8, c = 1$. The Lyapunov exponent diagram and bifurcation diagram of system (1) in Fig. 3.

**Figure 4:**
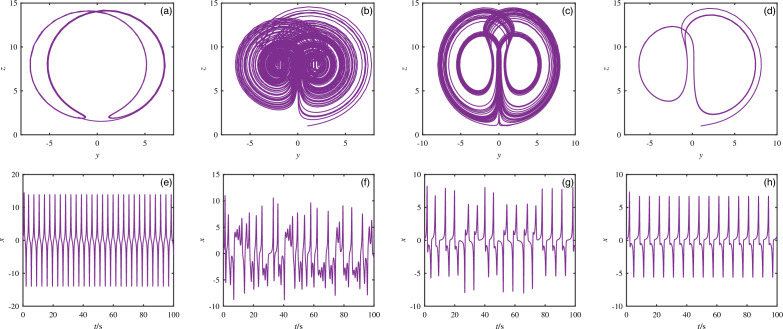
Attractor diagrams and timing diagrams for different periods. (**a**), (**e**) $a = 1$. (**b**), (**f**) $a = 4$. (**c**), (**g**) $a = 7.8$. (**d**), (**h**) $a = 9.5$.

Figure 3 shows system (1) is in a periodic state when parameters are $a\in [2,2.32]$; when choose parameters $a\in [2,2.32]$, $a\in [7,7.5]$ and $a\in [8.15,10]$, system (1) is in a period-doubling state; when parameters are $a\in [2.32,7]$ and $a\in [7.5,8.15]$, system (1) is in a chaotic state. Figure 4 depicts the motion trajectory of system (1) when taking values of different specific parameters through phase diagrams and timing diagrams. Table 2 describes the dynamic behavior trajectories in the corresponding period.

**Table 2:** System equilibrium stability.

Parameter	State	Location
$a=1$	Periodic	Fig. 4a, e
$a=4$	Chaos	Fig. 4b, f
$a=7.8$	Chaos	Fig. 4c, g
$a=9.5$	Period-doubling	Fig. 4d, h

**Figure 5:**
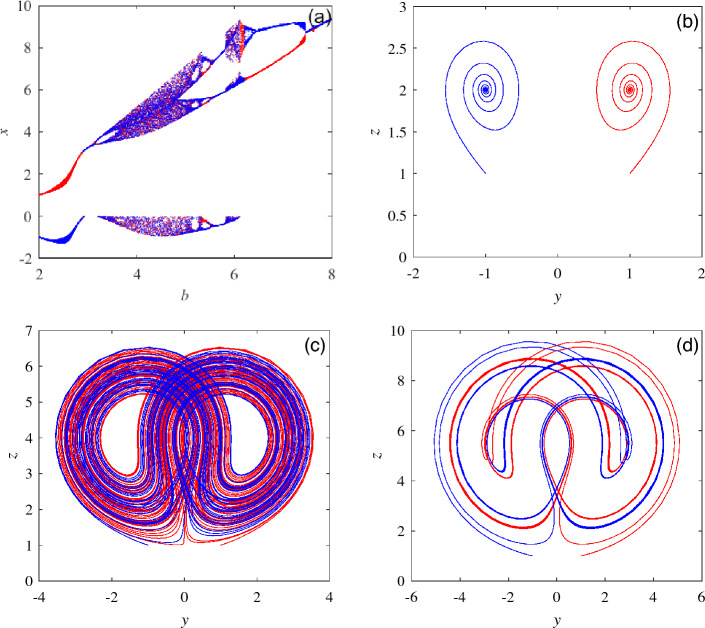
Coexistence of attractors under different parameter values. (**a**) The bifurcation diagram when the parameters are $a = 2, c = 1,b\in [2,8],$ and the initial values are $x_{01}=(1, 1, 1), x_{02}=(-1, -1, 1)$. (**b**) Attractor coexistence phenomenon when $b = 2$. (**c**) Attractor coexistence phenomenon when $b = 4$. (**d**) Attractor coexistence phenomenon when $b = 5.5$.

Chaotic systems with symmetric characteristics generally have the phenomenon of attractor coexistence^22^. To explore the coexistence of attractors in system (1), $a = 2, b\in [2,8], c = 1$ and the initial values $x_{01} = (1, 1, 1), x_{02} = (-1, -1, 1)$ are selected. The red is used to represent $x_{01}$ and blue is used to represent $x_{02}$. The bifurcation diagram of the system is shown in Fig. 5a. Fig. 5 and Table 3 show the coexistence of attractors in different periods.

**Table 3:** Coexistence of attractors in different periods.

Parameter	State	Location
$b=2$	Stable point coexistence	Fig. 5b
$b=4$	Coexistence of chaos attractors	Fig. 5c
$b=5.5$	Cycle doubling coexistence	Fig. 5d

By observing Fig. 5, system (1) has obvious attractor coexistence phenomenon under the selection of different initial values, and the dynamic behavior of system (1) is obviously different. By selecting specific value parameters, the static point coexistence phase diagram, chaotic attractor coexistence phase diagram and periodic attractor coexistence phase diagram of system (1) are obtained, and obvious coexistence phenomena can be observed.

## Variable structure characteristics of the system

On the basis of system (1), by changing the system structure, a new system model is obtained:

3 $$\begin{aligned} \left\{ \begin{array}{lll} \dot{x}=-ax+yz \\ \dot{y}=bx-xz \\ \dot{z}=-z+f(\cdot )+c \\ \end{array} \right. \end{aligned}$$

In Eq. (3), $f(\cdot )$ is a variable function body, which can occur the following possibilities:

4 $$\begin{aligned}{} & {} f(\cdot )= \left\{ \begin{array}{lll} kxy, \\ pxx, \\ kxy+pxx, \\ kxy+myy, \\ pxx+myy, \\ \end{array} \begin{array}{lll} 0<k<+\infty \\ 0<p<+\infty \\ 0<m<+\infty \\ \end{array} \right. \end{aligned}$$

5 $$\begin{aligned}{} & {} (k,p,m)= \left\{ \begin{array}{lll} k\ge {p},k<p, \\ k\ge {m},k<m, \\ p\ge {m},p<m, \\ \end{array} 0<k,p,m<+\infty \right. \end{aligned}$$

**Figure 6:**
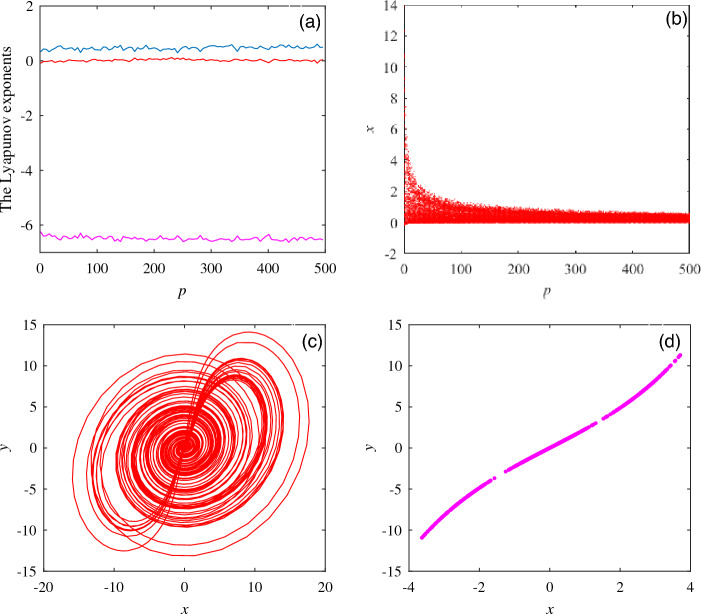
Related dynamic behavior. (**a**) The Lyapunov exponent diagram. (**b**) Bifurcation diagram. (**c**) Phase diagram. (**d**) Poincare section diagram.

Among them, *x*, *y* are system state variables, *k*, *p* and *m* are system unknown parameters. The values of *k*, *p* and *m* have the possibility of approaching positive infinity and the quantitative relationship in the Eq. (5) of *k*, *p* and *m*. When select $f(\cdot )= pxx$, to further show the influence of the values of variable *p* on the dynamic behavior of system (3), a wide range of intervals and unknown parameters *a *=5, b = 8, c = 1 and initial values (0.1, 0.1, 0.1) are selected to obtain the Lyapunov exponent diagram, bifurcation diagram, attractor diagram and Poincare section diagram of the system shown in Fig. 6.

The Lyapunov exponent diagram and bifurcation diagram in Fig. 6a and b show that system (3) has maintained chaotic characteristics in a large range. By comparing the attractor diagram at $p = 1$ with the Poincare section of *xy* plane at $p = 100, z = 4,$ the parameter *p* will change the existence range of chaotic attractor. With the increase of parameter *p*, the volume of attractor in *xy* plane will decrease. Fig. 6b and c show the images of corresponding periods.

**Figure 7:**
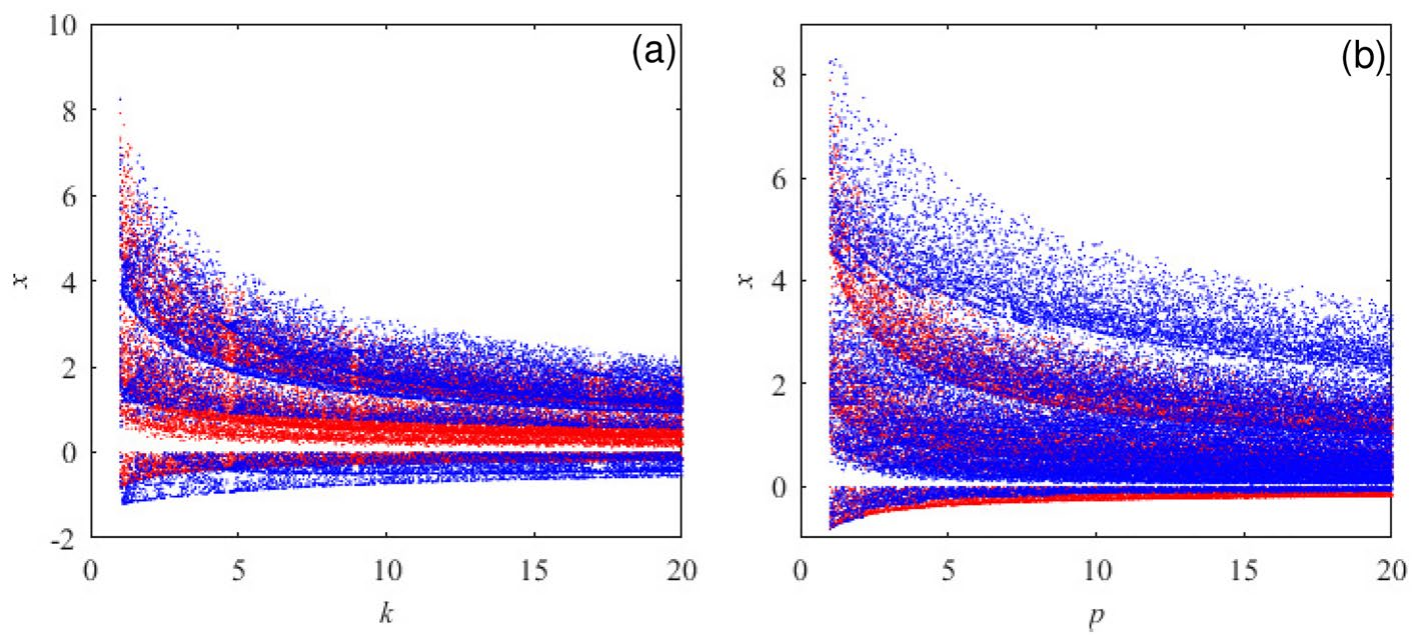
Comparison of bifurcation diagrams with different values of *k* and *p*. (**a**) Bifurcation diagram when $k > p$ and $k = p$. (**b**) Bifurcation diagram when $k < p$ and $k = p$.

When the nonlinear term is selected $f(\cdot )= kxy + pxx$, Eq. (5) shows that there are three possibilities of $f(\cdot )$ at this time, and these three situations are compared and expounded. When the relationship between *k* and *p* is $k>p$, $p=1,k\in [1,20]$ are selected to obtain the blue part in Fig. 7a of the bifurcation diagram of the system; when the relationship between *k* and *p* is $k = p$, the value ranges are selected as $k,p\in (1,20)$, and the red parts in Fig. 7a and b are obtained. When $k < p,k=1,p\in (1,20)$ are selected, the blue parts of the system bifurcation diagram in Fig. 7b are obtained. By comparing Fig. 7a and b to show the bifurcation diagrams in different periods.

**Figure 8:**
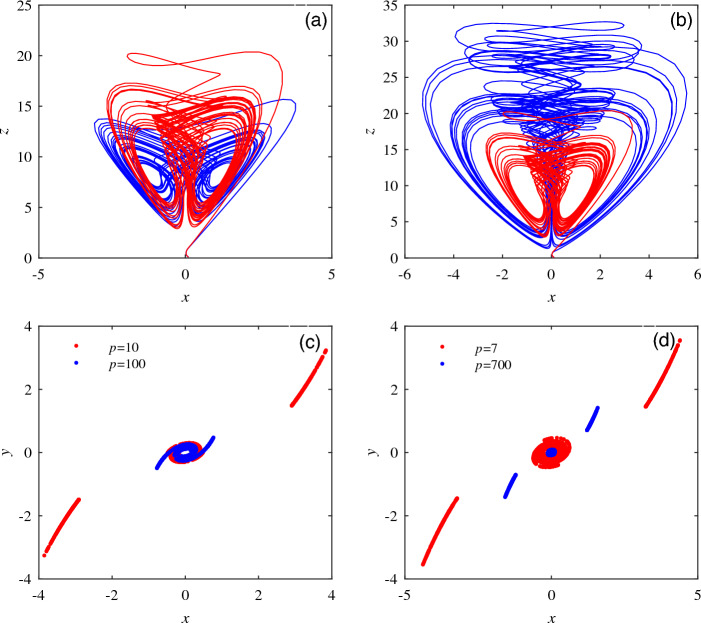
The cross sections of attractors and Poincare in different periods. (**a**) $k > p, p = 1, k = 7$ and $k = p = 7$ attractor coexistence diagram. (**b**) $k < p, k = 1, p = 7$ and $k = p = 7$ attractor coexistence diagram. (**c**) $k = p = 10$ and $k = p = 100, z = 14$ Poincare section diagram. (**d**) $k < p, k = 1, p = 7$ and $p = 700, z = 14$ Poincare section diagram.

To clearly show the difference of attractors in different periods, the numerical relation of *k* and *p* is $k > p$. The red chaotic attractor in Fig. 8a with $k = p = 7$ and the blue chaotic attractor in Fig. 8a with $p=1,k=7$ are obtained. The numerical relation of *k* and *p* is $k < p$. The red chaotic attractor in Fig. 8b with $k = p = 7$ and the blue chaotic attractor in Fig. 8b with $p=7,k=1$ are obtained. When $k = p = 10$ and $k = p = 100, z = 14$ are selected, the Poincare section of system (3) on the *xy* plane is shown in Fig. 8c; when $k < p, k = 1, p = 7$ and $p = 700, z = 14$ are selected, the Poincare section of system (3) on the *xy* plane is shown in Fig. 8d. The attractor diagram and Poincare section diagram have obvious changes when the values of *k* and *p* are different.

When the selections of $f(\cdot )$ are changed, the dynamic behaviors of system (3) are also constantly changing. There is a possibility that the values of nonlinear parameter in three-dimensional chaotic systems tend to be positively infinite. When the selections of $f(\cdot )$ are selected as other cases, system (3) will exhibit similar dynamic characteristics, which will not be further elaborated in this paper.

## Spectral entropy complexity analysis

**Figure 9:**
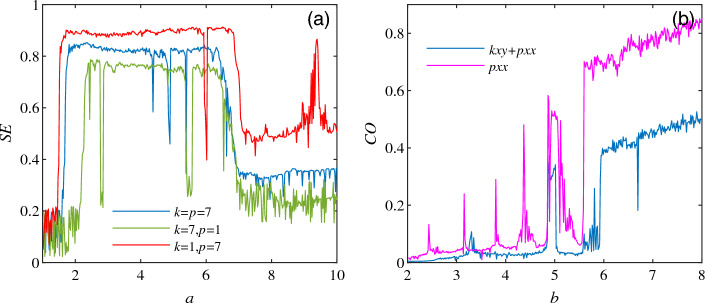
System (3) Comparison of complex dynamic characteristics. (**a**) Comparison of SE complexity when $f(\cdot )= kxy + pxx, k = p = 7$. (**b**) Comparison of C0 complexity when $f(\cdot )= kxy + pxx$ and $f(\cdot )= pxx, k = p = 7$.

Complexity measurement provides a certain analysis basis for studying the dynamic behavior of the system. In this paper, the frequency domain complexity measurement algorithm C0 and SE algorithm^23,24^ are used to explore the system complexity when the switched system (3) is at $f(\cdot )=kxy+pxx$, $0<k,p<+\infty$. When the initial conditions of system (3) are selected as $a\in [1,10]$, $b=8, c=1$ and the initial value of (0.1, 0.1, 0.1), the quantitative relationship between *k* and *p* is compared, and the comparison of complex dynamic characteristics of the system are shown in Fig. 9a. When the switched system (3) is at $f(\cdot )=kxy+pxx$ and $f(\cdot )=pxx$ and $k=p=7$, the parameters $a =5,b\in [2,8],c=1$ are selected, and the comparison of dynamic complex characteristics of the system are shown in Fig. 9b.

Figure 9a shows the values of parameter *k* and *p* are different in the complex characteristic curve of system (3), the complexity of the system is significantly different. Figure 9b shows that the complexity of system (3) varies considerably with the selection of $f(\cdot )$.

## NIST test

To verify the pseudo-randomness of the random sequence of the chaotic system under the condition of large-scale parameters in system (1), this paper uses the 15 test methods given in SP800 - 22 Revision 1a^25^ to test the random characteristics of the bit sequence of the chaotic system. Among them, 15 tests each item produce the P-value values and compare it with the given level to determine whether the sequence of the system is random. When the value of the generated P-value $\ge$0.01, the sequence of the system is random. Otherwise, the sequence of the system is not random. The length of the bit sequence recommended in SP800-22 Revision 1a is $10^2$ to $10^7$ and the length of the test series *S* selected in this paper is $n=10^6$. Equation (6) is the discretization form of Runge-Kutta method of system (1), parameters are selected as $a = 5, b = 8, c = 1, p = 7$ and $p = 700$. The initial values $(x_0,y_0,z_0)=(1.1,2.2,3.3)$ are selected and 100 million bits data are generated. Table 4 shows the corresponding test data.

6 $$\begin{aligned} \left\{ \begin{array}{lll} x_{i+1}=x_i+\frac{h}{6}(K_{11}+2K_{12}+2K_{13}+K_{14})\\ K_{11}=y_iz_i-ax_i\\ K_{12}=y_iz_i-a(x_i+\frac{h}{2}K_{11})\\ K_{13}=y_iz_i-a(x_i+\frac{h}{2}K_{12})\\ K_{14}=y_iz_i-a(x_i+hK_{13})\\ y_{i+1}=y_i+\frac{h}{6}(K_{21}+2K_{22}+2K_{23}+K_{24})\\ K_{21}=bx_{i+1}-x_{i+1}z_i\\ K_{22}=bx_{i+1}-x_{i+1}z_i\\ K_{23}=bx_{i+1}-x_{i+1}z_i\\ K_{24}=bx_{i+1}-x_{i+1}z_i\\ z_{i+1}=z_i+\frac{h}{6}(K_{31}+2K_{32}+2K_{33}+K_{34})\\ K_{31}=px_{i+1}y_{i+1}-z_i+c\\ K_{32}=px_{i+1}y_{i+1}-(z_i+\frac{h}{2}K_{31})+c\\ K_{33}=px_{i+1}y_{i+1}-(z_i+\frac{h}{2}K_{32})+c\\ K_{34}=px_{i+1}y_{i+1}-(z_i+hK_{33})+c\\ \end{array} \right. \end{aligned}$$

**Table 4:** NIST test results.

No.	Test method	P-value of *p* = 7	P-value of *p* = 700	Result
1	Frequency	0.9662	0.4284	Success
2	Block frequency	0.6008	0.9135	Success
3	Runs	0.3506	0.2598	Success
4	Longest run	0.2968	0.0559	Success
5	Rank	0.2038	0.8625	Success
6	FFT	0.1687	0.0348	Success
7	Nonoverlapping template	0.6077	0.0340	Success
8	Overlapping template	0.3233	0.4298	Success
9	Universal	0.5587	0.3234	Success
10	Linear vomplexity	0.3752	0.2587	Success
11	Serial	0.4472	0.1636	Success
12	Approximate entropy	0.0441	0.5946	Success
13	Cumulative dums	0.9971	0.9374	Success
14	Random excursions	0.4083	0.8896	Success
15	Random excursions variant	0.9619	0.4224	Success

Table 4 shows when the values of *p* are expanded by 100 times.The values of the P-value obtained by the test all satisfy P-value $\ge$0.01, which proves that the random sequence of the system has excellent pseudo-randomness.

## The existence of toggle terms in unified chaotic systems

In 2002, Lü and Chen proposed to connect the Lorenz system with the Chen system, proposing the unified chaotic system^26^. The proposal of unified chaotic systems reveals the relationship between chaotic systems with similar structures, which provides an important theoretical basis for the study of chaotic systems with similar chaotic characteristics. The chaotic model of a unified chaotic system is:

7 $$\begin{aligned} \left\{ \begin{array}{lll} \dot{x}=(25a+10)(y-x)\\ \dot{y}=(28-35a)x-xz+(29a-1)y\\ \dot{z}=xy-z(a+8)/3\\ \end{array} \right. \end{aligned}$$

When the parameter value range of the unified chaotic system is $a\in [0,1]$, the system will transition from a generalized Lorenz system to a generalized Chen system, and the system has been in a chaotic state. When $a\in [0,0.8)$, the system belongs to the generalized Lorenz system; when $a=0.8$, the system belongs to the generalized Lü system; when $a\in (0.8,1]$, the system belongs to the generalized Chen system.

The nonlinear term *xy* in Eq. (7) is replaced with the switchable branch of $f(\cdot )$ in Eq. (4), and the switchable branch proposed in this paper is applied to the unified chaotic system. To investigate the changes in the dynamic behavior of unified chaotic systems under different initial conditions.

8 $$\begin{aligned} \left\{ \begin{array}{lll} \dot{x}=(25a+10)(y-x)\\ \dot{y}=(28-35a)x-xz+(29a-1)y\\ \dot{z}=f(\cdot )-z(a+8)/3\\ \end{array} \right. \end{aligned}$$

Calculate dissipation $\nabla {V}$ for system (8):

9 $$\begin{aligned} \nabla {V}=\frac{\partial {\dot{x}}}{\partial {x}}+\frac{\partial {\dot{y}}}{\partial {y}}+\frac{\partial {\dot{z}}}{\partial {z}}=(11a-41)/3 \end{aligned}$$

The dissipation degree $\nabla {V}$ in Eq. (9) that the dissipation degree of system (8) is affected by the value of parameter *a*. When $a\in [0,1]$, system (8) is still a dissipative system and converges with exponential $(e^{(11a-41)/3}\mathrm{{dV}})/\mathrm{{dt}}$, indicating that the volume of system (8) at *t* time is contracted from $V_0$ to $V_0e^{(11a-41/3)}$.

At time $t\rightarrow \infty$, the volume element of system (8) converges exponentially to 0, enabling the system to produce bounded attractors, indicating that switching branch $f(\cdot )$ has no effect on the dissipative properties of system (8), and eventually produces bounded attractors.

To specifically show the influence of the selection of switching branch $f(\cdot )$ on the dynamic behavior of unified chaotic systems, different switching branches in $f(\cdot )$ are selected as examples to show the dynamic behavior transformation of system (8) from the Lyapunov exponent diagram and the Poincare section diagram.

**Figure 10:**
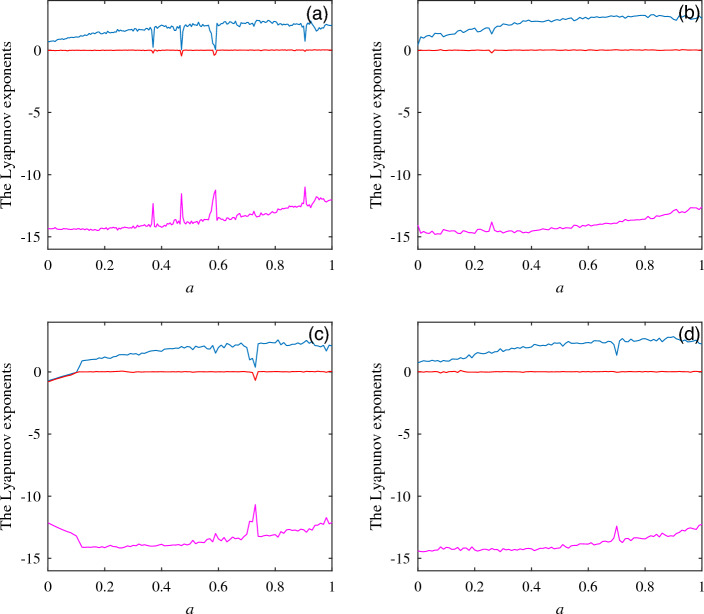
The Lyapunov exponent diagram when switching branches are selected.

**Figure 11:**
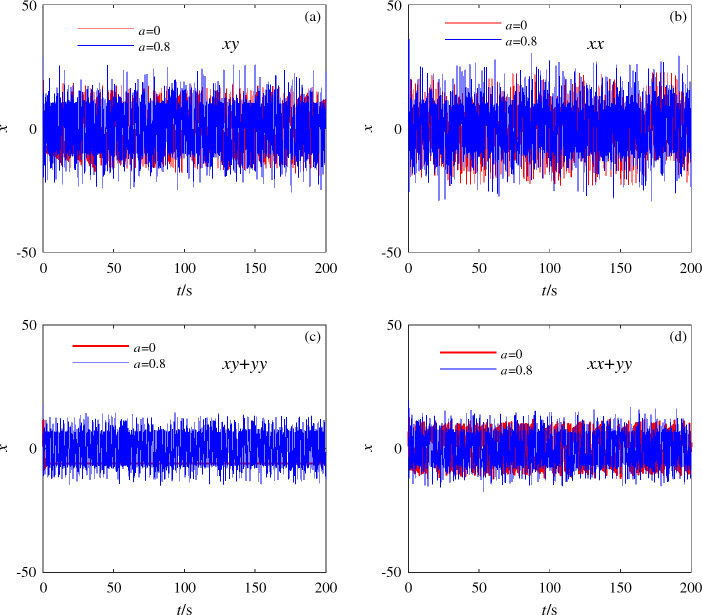
Timing diagrams of system (8) under the condition of different parameter values.

**Figure 12:**
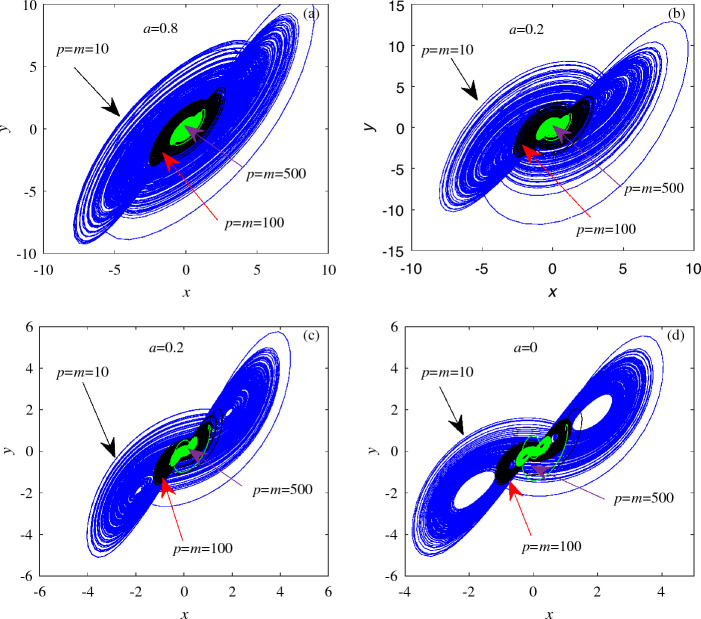
The existence of chaotic attractors in system (8) under the condition of large parameter values of different switching branches.

The initial value in the unified chaotic system (8) is selected as (1,1,1), and the values of *k*, *p*, *m* in the switching option are set to $k=p=m=1$. The Lyapunov exponent diagrams of system (8) under different switching conditions are obtained, as shown in Fig. 10. To observe more clearly the influence of the selection of different switching branches on the chaotic characteristics of the unified chaotic system, the existence of dynamic behavior is compared and analyzed by using the timing diagrams, as shown in Fig. 11. At the same time, the value range of the switching branch in the unified chaotic system is explored, and the existence of attractors in the unified chaotic system under the condition of a wide range of unknown parameters is observed. Fig. 12 shows the comparison of attractors with a wide range of values for different switching branches under the same initial conditions. Table 5 is used to systematically demonstrate the effect of switching branch $f(\cdot )$ selection on the dynamic behavior of chaotic systems.

**Table 5:** Influence of switching branch $f(\cdot )$ on the dynamic behavior of unified chaotic systems.

$f(\cdot )$	*kxy*	*pxx*	$kxy+myy$	$pxx+myy$
Lyapunov exponential diagram	Fig. 10a	Fig. 10b	Fig. 10c	Fig. 10d
Timing diagram	Fig. 11a	Fig. 11b	Fig. 11c	Fig. 11d
Phase diagram	Fig. 12a	Fig. 12b	Fig. 12c	Fig. 12d

By observing the Lyapunov exponent diagram under different switching branch conditions in Fig. 10, different branch conditions have an impact on the chaotic characteristics of the unified chaotic system. In the value interval of parameter $a\in [0,1]$, several switching branches produce dynamic behaviors such as static point, period, and chaos at the same time, indicating that the switching branch proposed in this paper also produces better application effects when applied to unified chaotic systems.

The change of motion trajectory in a single dimension in Fig. 11 reveals the influence of switching branches on the motion trajectory of attractors in unified chaotic systems. It shows that the unified chaotic system also has the characteristics of chaotic system being extremely sensitive to the initial state after applying switch branch $f(\cdot )$. Explain the sensitivity of system (8) to the initial value. Since the third chapter of this paper, different switching branches applied to system (3) exhibit a wide range of chaotic characteristics. Fig. 12 shows the large-scale chaotic properties existing in unified chaotic system (8). The random value method is used to show the difference in the motion trajectory of unknown parameters in a wide range of value ranges. By using different research methods for the dynamic behavior of different switching branches on unified chaotic systems in Table 5, the applicability of the switching options proposed in this paper in unified chaotic systems is fully demonstrated.

## Circuit simulation design

**Figure 13:**
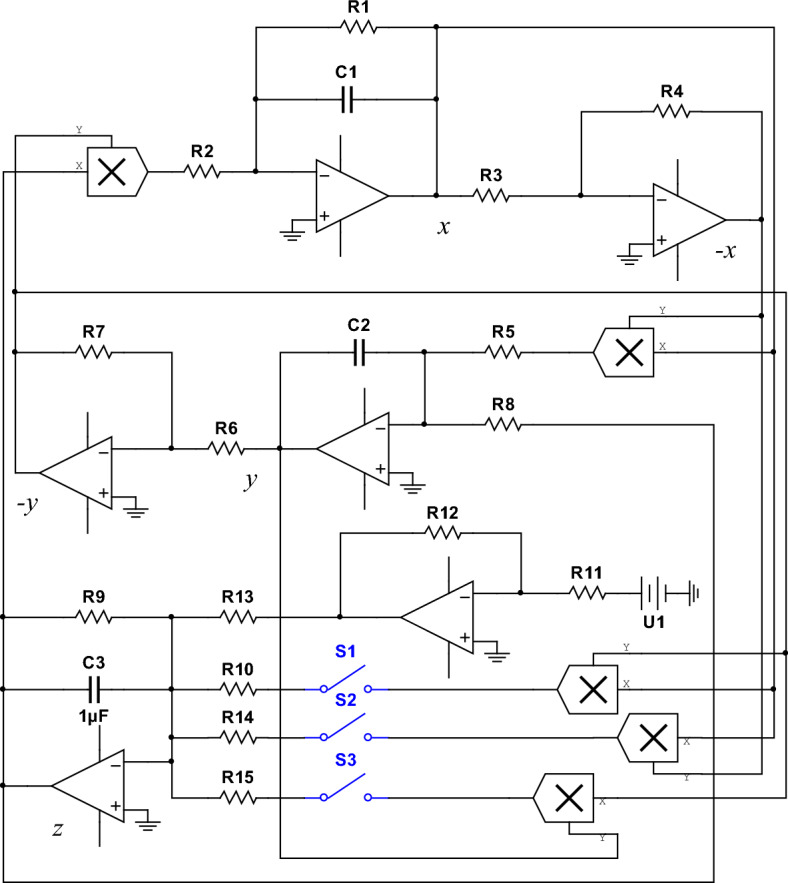
Circuit schematic diagram of system (1) and (3).

To verify the feasibility of system (1) and (3) circuit implementation, Multisim simulation software is used to build the simulation circuit diagram Fig. 13 of the system.

The output gain of the multiplier (AD633) in Fig. 13 is 1. The operational amplifier (LM324M), selector switch and other related components are used for addition, subtraction, integration and other related operations. Apply Kirchhoff law to Fig. 13 to get the differential equation:

10 $$\begin{aligned}{} & {} \left\{ \begin{array}{lll} \frac{\mathrm{{d}}x}{\mathrm{{d}}t}=-\frac{1}{C_1R_1}x+\frac{R_7}{C_1R_2R_6}yz\\ \\ \frac{\mathrm{{d}}y}{\mathrm{{d}}t}=\frac{R_4}{C_2R_3R_5}x-\frac{1}{C_2R_8}xz\\ \\ \frac{\mathrm{{d}}z}{\mathrm{{d}}t}=-\frac{1}{C_3R_9}z+\frac{R_{12}}{C_3R_{11}R_{13}}\textrm{U1}-\omega \end{array} \right. \end{aligned}$$

11 $$\begin{aligned}{} & {} \omega = \left\{ \begin{array}{lll} -\frac{R_7}{C_3R_6R_{10}}xy\\ \\ -\frac{R_4}{C_3R_3R_{14}}x^2\\ \\ -\frac{R_7}{C_3R_6R_{15}} \end{array} \right. \end{aligned}$$

**Figure 14:**
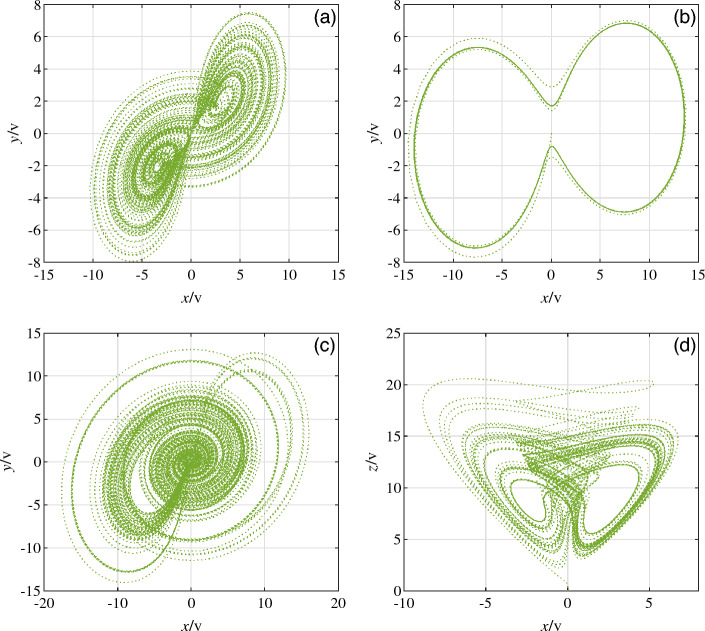
Circuit simulation attractor (**a**) Attractor diagram for $a = 5$ and a nonlinear term of *xy* (**b**) Attractor diagram for $a = 1$ and a nonlinear term of *xy* (**c**) Attractor diagram for $a = 5$ and a nonlinear term of $x^2$ (**d**) Attractor diagram for $a = 5$ and a non-linear term of $x^2+xy$.

By using the switch to select the different nonlinear terms connected to the circuit, chaotic systems with different combinations of nonlinear terms can be obtained by varying the resistor values. The chaotic systems are selected when the nonlinear terms at the variable $\omega$ are $xy, x^2$ and $xy+x^2$, the corresponding attractor diagrams are obtained by varying the resistance values. When the parameters $b=8, c=1, a=5$ and $a=1$ are chosen for system (1), the attractor diagrams Fig. 14a and b are obtained from the circuit simulation by adjusting the resistance of $R_1$. Equation (3) is compared with Eq. (10) when the unknown parameters are chosen as $a=5, b=8, c=1, p=1, k=1$ and initial values of (0.1, 0.1, 0.1).

12 $$\begin{aligned}{} & {} \left\{ \begin{array}{lll} \frac{1}{C_1R_1}=5,\frac{R_7}{C_1R_2R_6}=1\\ \\ \frac{R_4}{C_2R_3R_{5}}=8,\frac{1}{C_2R_8}=1\\ \\ \frac{1}{C_3R_9}=1,\frac{R_{12}}{C_3R_{11}R_{13}}=1,\mathrm{{U}_1}=1\\ \\ \frac{R_7}{C_3R_6R_{10}}=1,\frac{R_4}{C_3R_3R_{14}}=1 \end{array} \right. \end{aligned}$$

13 $$\begin{aligned}{} & {} \left\{ \begin{array}{lll} R_1=200\mathrm{{k\Omega }},R_2=1000\mathrm{{k\Omega }},R_5=125\mathrm{{k\Omega }}\\ R_{11}=100\mathrm{{k\Omega }},R_8=1000\mathrm{{k\Omega }},R_9=1000\mathrm{{k\Omega }}\\ R_{10}=1000\mathrm{{k\Omega }},R_{12}=10\mathrm{{k\Omega }},R_{13}=100\mathrm{{k\Omega }}\\ R_{14}=1000\mathrm{{k\Omega }},R_3=R_4=R_6=R_7=100\mathrm{{k\Omega }} \end{array} \right. \end{aligned}$$

The attractor diagram of system (3) corresponding to *xy* is shown in Fig. 14a and b, the attractor diagram of $x^2$ is shown in Fig. 14c and the attractor diagram of $xy+x^2$ is shown in Fig. 14d.

The attractor diagrams under different simulations shown in Fig. 14 are consistent with the trajectories of the attractor diagrams in Figs. 1 and 4, indicating that the simulation results obtained by circuit simulation are successful. This paper provides theoretical support for applying system (3) proposed in this paper to hardware implementation.

## Physical implementation

In practical application, due to device error and other reasons, the simulation results cannot be guaranteed to be consistent with the actual experiment. Therefore, it is necessary to verify the physical circuit of the chaotic system.

**Figure 15:**
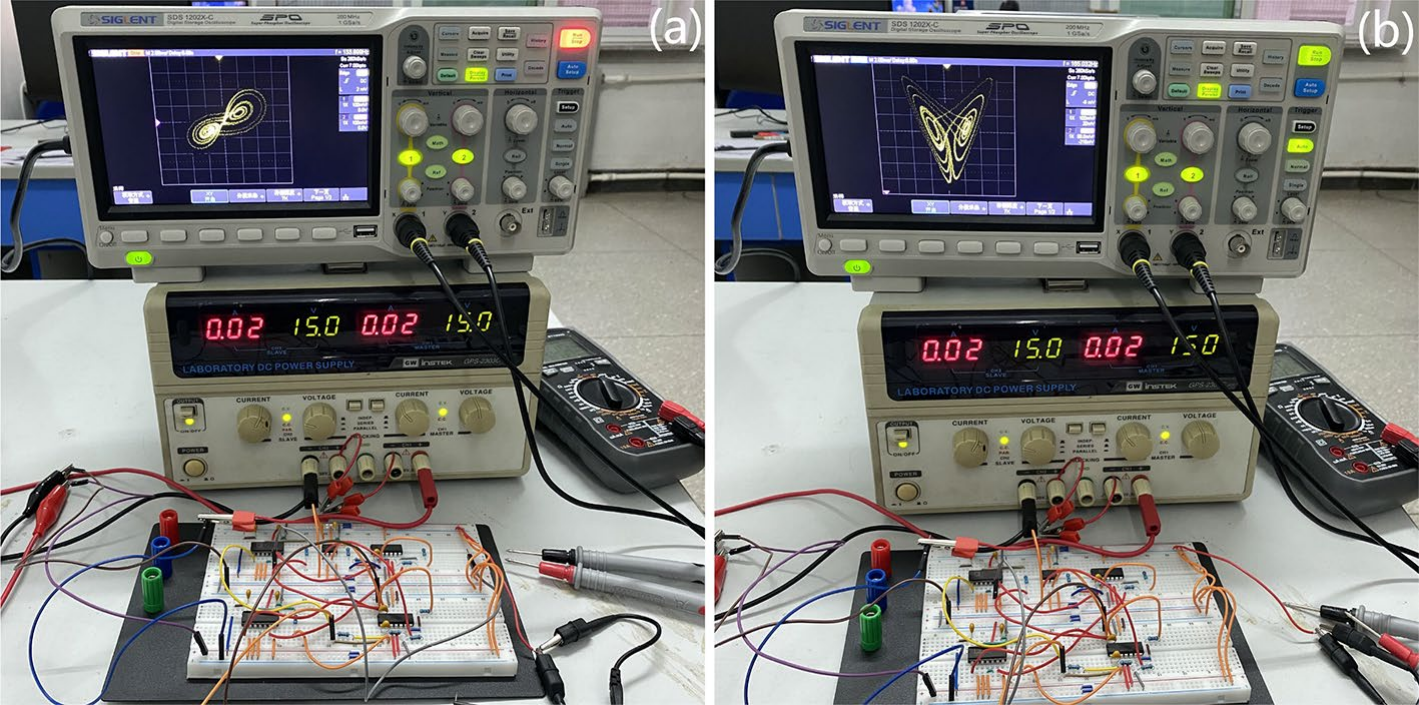
Breadboard schematic (**a**) $x-z$ phase diagram of system (1) (**b**) $x-y$ phase diagram of system (1).

The output gain of the multiplier (AD633) in Fig. 15 is 0.1. The operational amplifier (LM324M), Fig. 15 shows the physical implementation of the designed chaotic circuit system (1)on a breadboard. The $x-z$ attractor diagram of chaotic system (1) is shown in Fig. 15a, and the $x-y$ attractor diagram of system (1) is shown in Fig. 15b.The hardware circuit on the breadboard is composed of 3 analog multipliers AD633, 2 operational amplifiers LM324N, 9 capacitors and 11 resistors.

**Figure 16:**
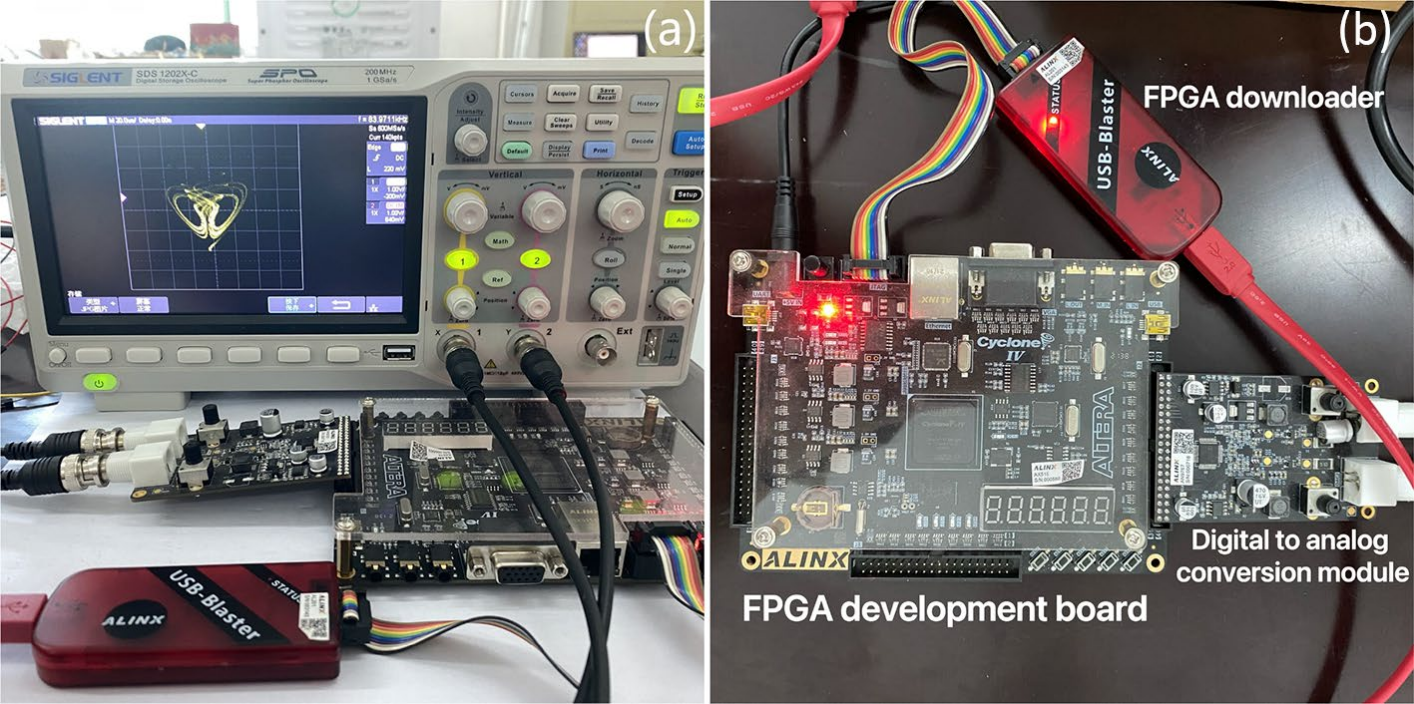
Physical realization diagram (**a**) Hardware rendering of FPGA implementation (**b**) Hardware diagram of FPGA.

The switchable chaotic system is implemented using FPGA technology. The hardware implementation involves several key components, namely the FPGA development board, the AN9767 black gold dual-channel 14-bit DA output module, a downloader, and an oscilloscope. The hardware setup is depicted in Fig. 16, where Fig. 16(a) represents the hardware effect diagram of the FPGA implementation, and Fig. 16b illustrates the hardware diagram of the FPGA.

**Figure 17:**
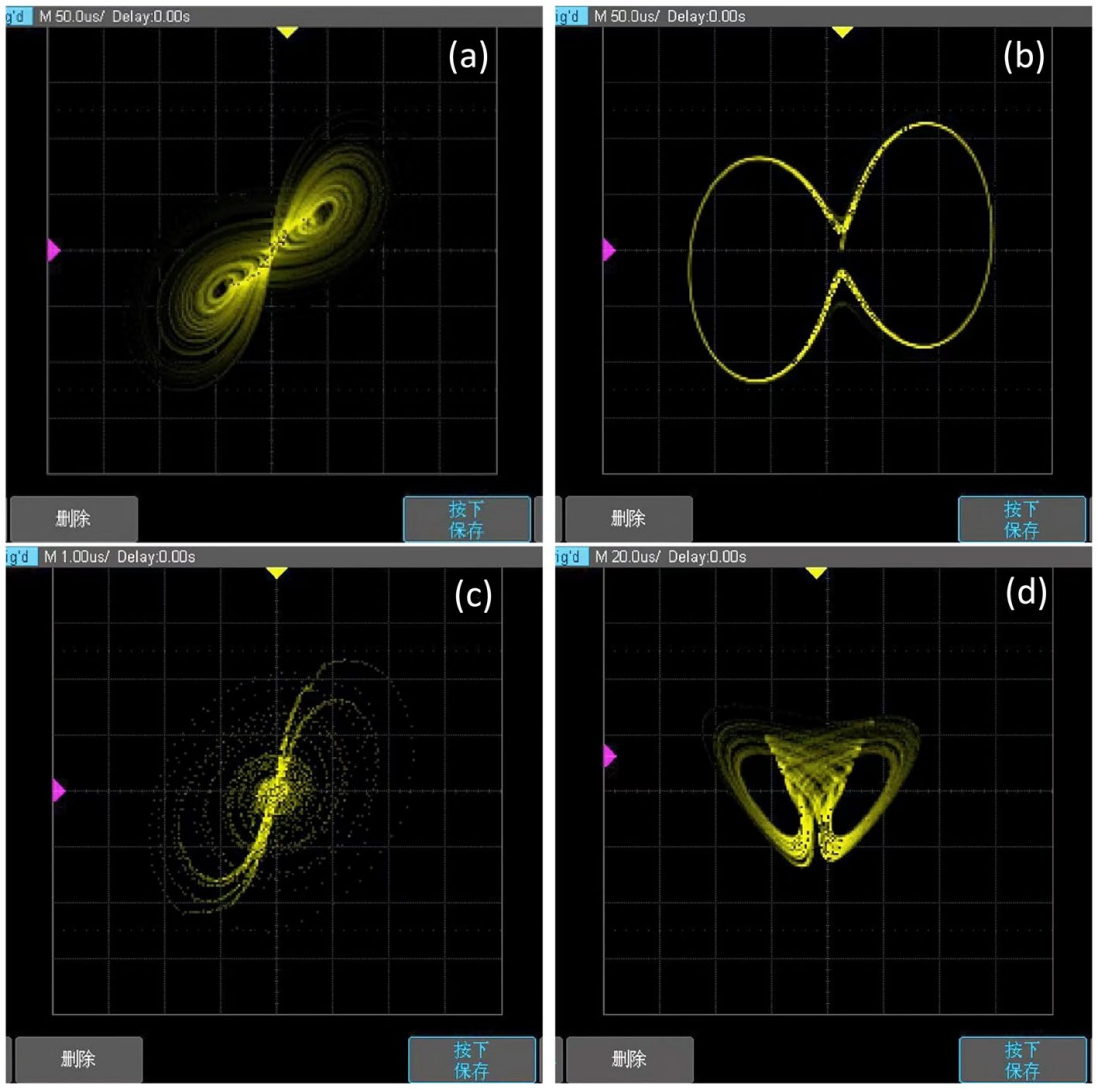
FPGA physical implementation diagram (**a**) Attractor diagram for $a = 5$ and a nonlinear term of *xy* (**b**) Attractor diagram for $a = 1$ and a nonlinear term of *xy* (**c**) Attractor diagram for $a = 5$ and a nonlinear term of $x^2$ (**d**) Attractor diagram for $a = 5$ and a non-linear term of $x^2+xy$.

The result diagram of FPGA hardware implementation of the switching system is shown in Fig. 17. The attractor diagram of the system (3) corresponding to *xy* is shown in Fig. 17a and b, while the attractor diagram of the system corresponding to $x^2$ is shown in Fig. 17c. Additionally, the attractor diagram of the system corresponding to $xy + x^2$ is shown in Fig. 17d.

## Conclusion

Under the condition of variable parameters, this study fully demonstrates the existence of attractors at different times and illustrates the coexistence of attractors in chaotic systems when the initial value is symmetrical. The switching system exhibits different chaotic characteristics depending on the selection of nonlinear terms and parameters. The application of the switchable method to the unified chaotic system has a significant effect. The chaotic sequence generated by the system is tested by expanding the parameters by a hundred times. Additionally, a simulation circuit for the switching system is designed and physically implemented.

## Data Availability

The data that support the findings of this study are available within the article. Further requests can be made to the corresponding author.
